# Supplementary material for: Discovery of Paralogous GnRH and Corazonin Signaling Systems in an Invertebrate Chordate
Source: Genome Biol Evol. 2023 Jun 9;15(7):evad108. doi: 10.1093/gbe/evad108 (PMC10321317; doi:10.1093/gbe/evad108)
Supplement: evad108_Supplementary_Data [file evad108_supplementary_data.zip › Supplementary Figures.pdf]

Supplementary Figures

Discovery of paralogous GnRH and corazonin signaling systems in an invertebrate chordate

Luis Alfonso Yañez Guerra and Meet Zandawala

atg	gct	gcg	aga	ctc	ccc	gca	ctt	ctt	gcc	gtc	ctg	ctg	ttg	gca	caa	atc	ctg	tgt	gcc
**M**	**A**	**A**	**R**	**L**	**P**	**A**	**L**	**L**	**A**	**V**	**L**	**L**	**L**	**A**	**Q**	**I**	**L**	**C**	**A**
cga	gcc	ttc	acc	tac	acg	cac	acg	tgg	ggc	cgc	aag	cgc	gca	gac	tcg	tca	gag	ctg	ctc
**R**	**A**	**F**	**T**	**Y**	**T**	**H**	**T**	**W**	**G**	**R**	**K**	**R**	**A**	**D**	**S**	**S**	**E**	**L**	**L**
aca	ccg	cat	gct	gcc	gcg	gac	agc	gtc	tct	gca	gcg	gag	gtt	tac	gac	gca	agt	gaa	ggg
**T**	**P**	**H**	**A**	**A**	**A**	**D**	**S**	**V**	**S**	**A**	**A**	**E**	**V**	**Y**	**D**	**A**	**S**	**E**	**G**
tca	gag	gtc	acc	aaa	gaa	gac	ttc	aag	atg	gct	gtt	cgt	act	ttg	ttc	cgg	atc	ctg	ggg
**S**	**E**	**V**	**T**	**K**	**E**	**D**	**F**	**K**	**M**	**A**	**V**	**R**	**T**	**L**	**F**	**R**	**I**	**L**	**G**
gac	tac	ctc	cag	aaa	agg	acg	aac	cag	aac	tga									
**D**	**Y**	**L**	**Q**	**K**	**R**	**T**	**N**	**Q**	**N**	**-**									

Figure S1: *Branchiostoma floridae* CRZ cDNA sequence (Accession no: KF601546.1) and the deduced amino acid sequence. Within the amino acid sequence, the signal peptide is highlighted in blue, the mature peptide is in red and the predicted cleavage site is in green. The residues in pink within the signal peptide were part of the putative CRZ mature peptide predicted previously by Roch et al. (2014).

```
atg aaa acg ctg ttg ata tca ctg tcc att gtg ctg gtc ctg ctc cac acg tcc gag gcc
 M   K   T   L   L   I   S   L   S   I   V   L   V   L   L   H   T   S   E   A
tac tcg tac tcc tac ggg ttc gcg cca gga aag aaa aga gcc gcc ttg ctc gga gag aag
 Y   S   Y   S   Y   G   F   A   P   G   K   K   R   A   A   L   L   G   E   K
atc cga agt ctg cta cat aaa cac aga aac cag tac agt ccc gag gga acg acc aca cag
 I   R   S   L   L   H   K   H   R   N   Q   Y   S   P   E   G   T   T   T   Q
tct atg gat gat gag aca cca cgg ggc cca tcg acg tat ccc ttc ctc gtg aac gaa gcg
 S   M   D   D   E   T   P   R   G   P   S   T   Y   P   F   L   V   N   E   A
gaa ccc aag ggc cag gac acg tgg tac tcc gag cca atg tgg gga gca tct cgt gga gcc
 E   P   K   G   Q   D   T   W   Y   S   E   P   M   W   G   A   S   R   G   A
acg cga gac tac aac cct gcc ttg cgc agc tgg att tct gac aga agg aag tga
 T   R   D   Y   N   P   A   L   R   S   W   I   S   D   R   R   K   -
```

Figure S2: *Branchiostoma floridae* GnRH cDNA sequence (Accession no: XM_035819417.1) and the deduced amino acid sequence. Within the amino acid sequence, the signal peptide is highlighted in blue, the mature peptide is in purple and the predicted cleavage site is in green.

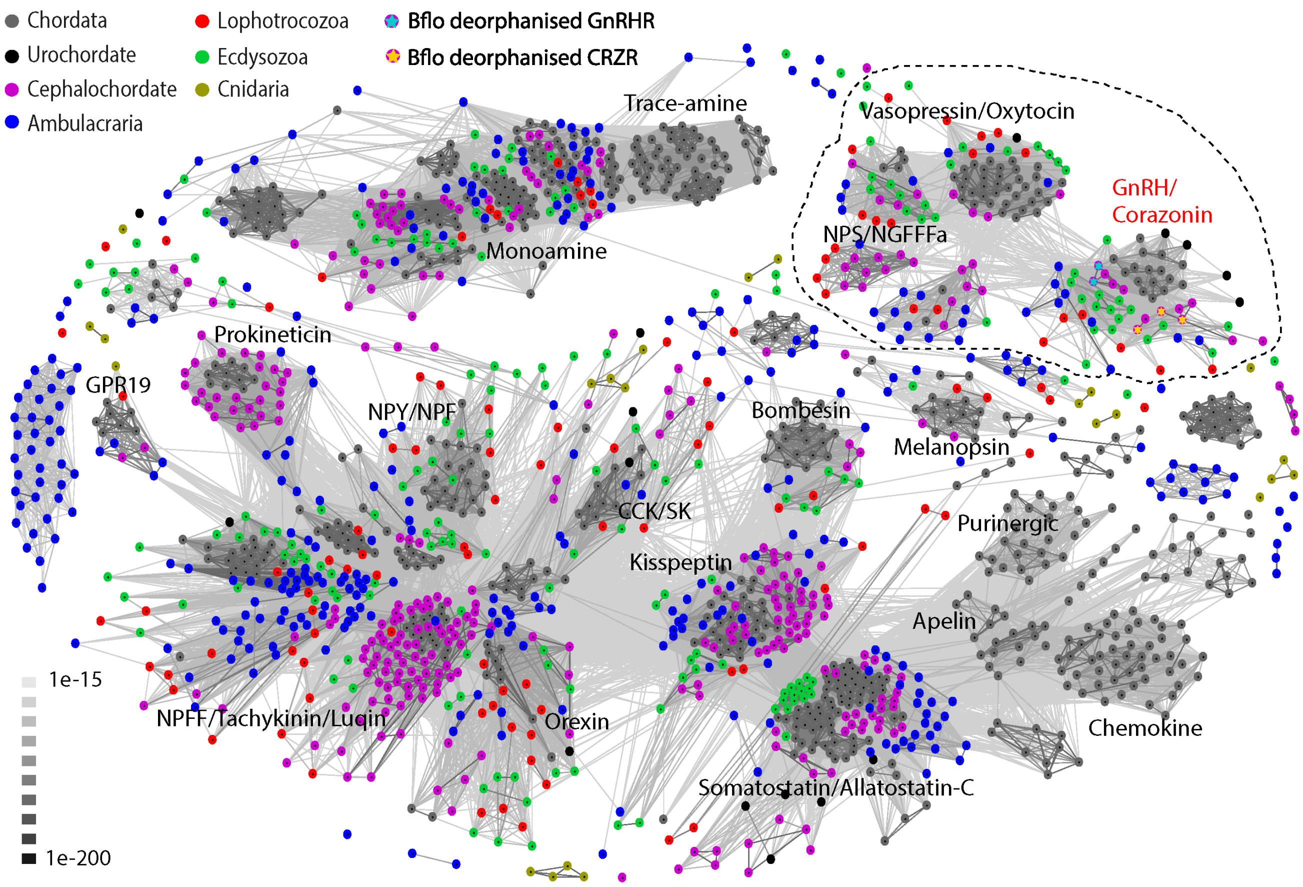

Figure S3: Cluster analysis of GPCRs shows that GnRH and CRZ receptors cluster closely with vasopressin/oxytocin and Neuropeptide-S (NPS)/NGFFFa receptors (dotted region). Each dot represents an individual receptor which has been color-coded according to its phyla. The receptors in the dotted region were used for the phylogenetic analysis in Figure 2A. The three CRZ receptors and two GnRH receptors from *Branchiostoma floridae* characterized in this study have been marked with yellow and cyan stars, respectively. Edges represent BLAST connections of *P* value < 1e-15.

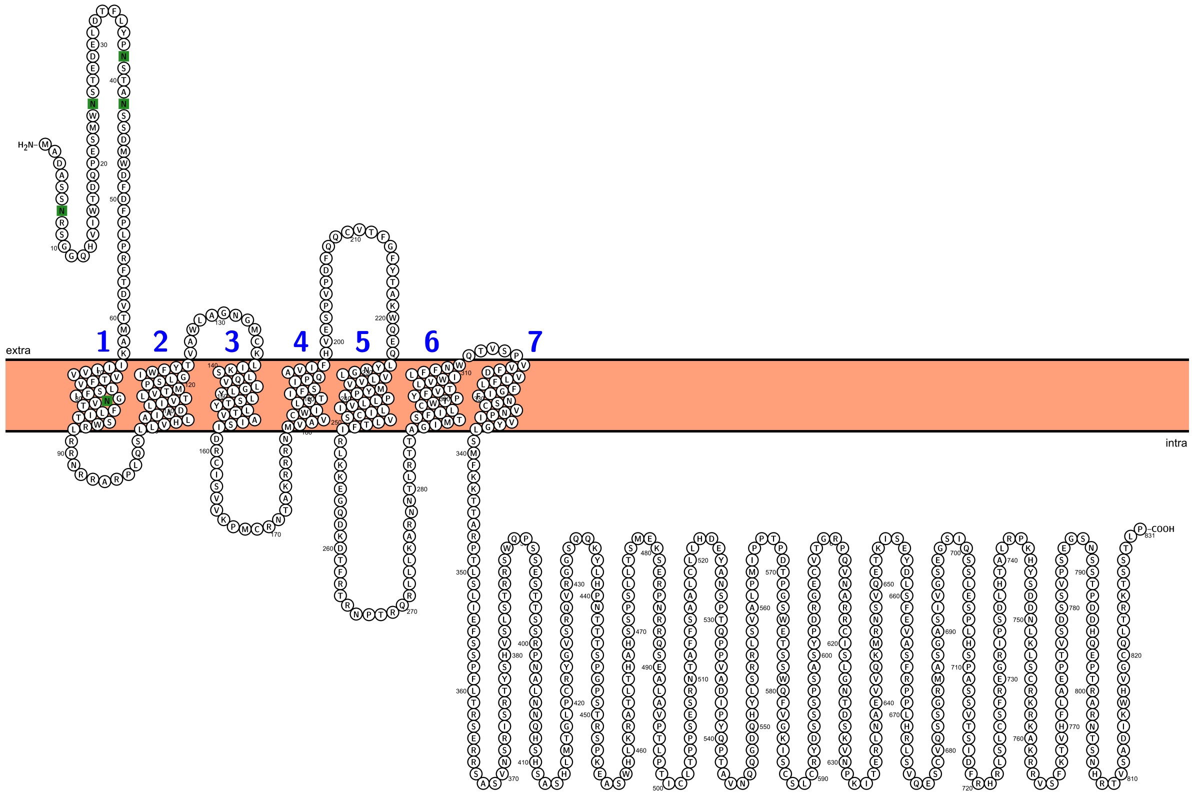

Figure S4: *In silico* prediction of the *Branchiostoma floridae* CRZR1 topology. The transmembrane domains are numbered successively in blue and the putative N-glycosylation sites are shown with green boxes.

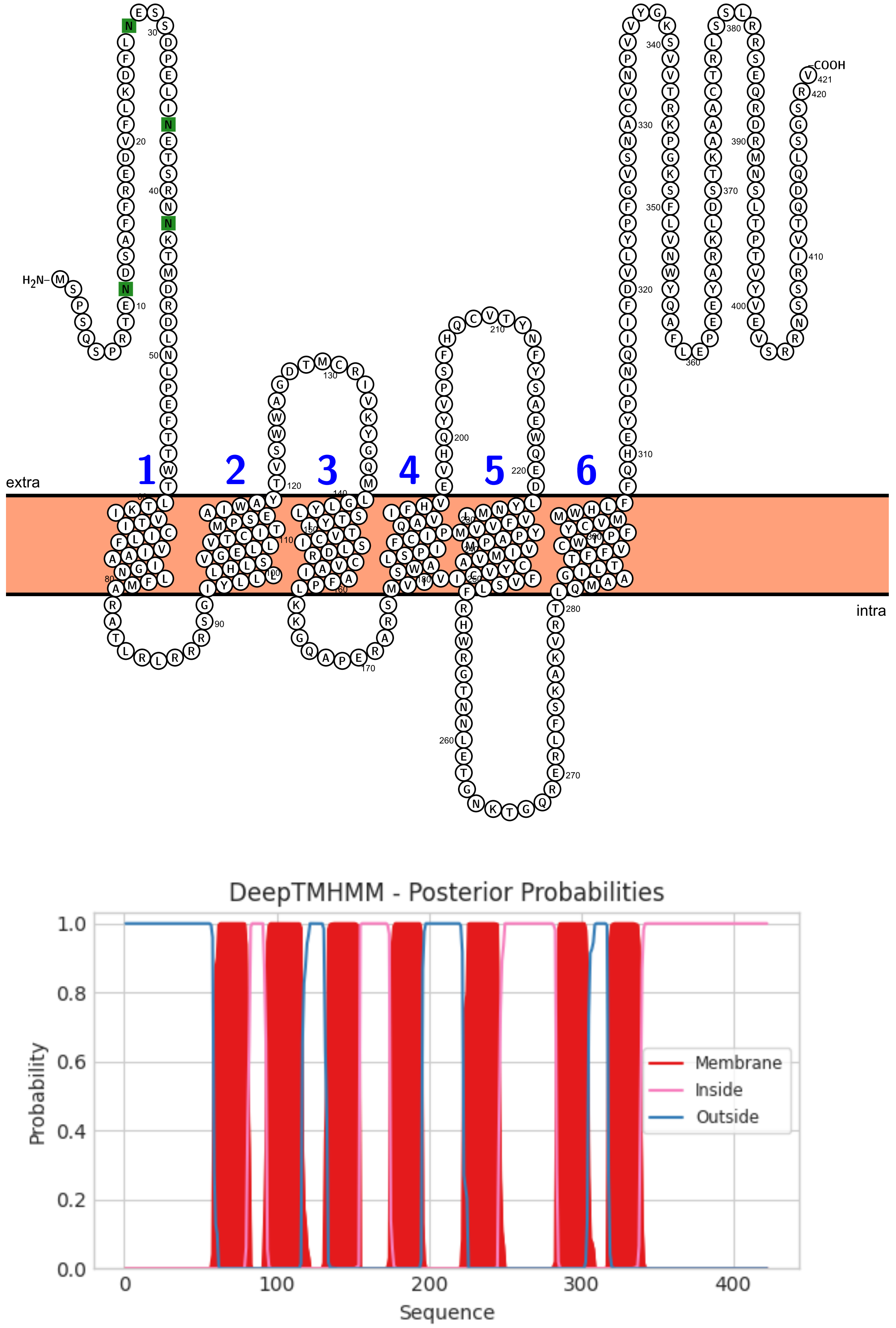

Figure S5: *In silico* prediction of the *Branchiostoma floridae* CRZR2 topology using Protter (top) and DeepTMHMM (bottom). For the prediction using Protter, the transmembrane domains are numbered successively in blue and the putative N-glycosylation sites are shown with green boxes. Since GPCRs typically have 7 transmembrane domains, the topology predicted by DeepTMHMM appears to be more accurate than Protter for this receptor.

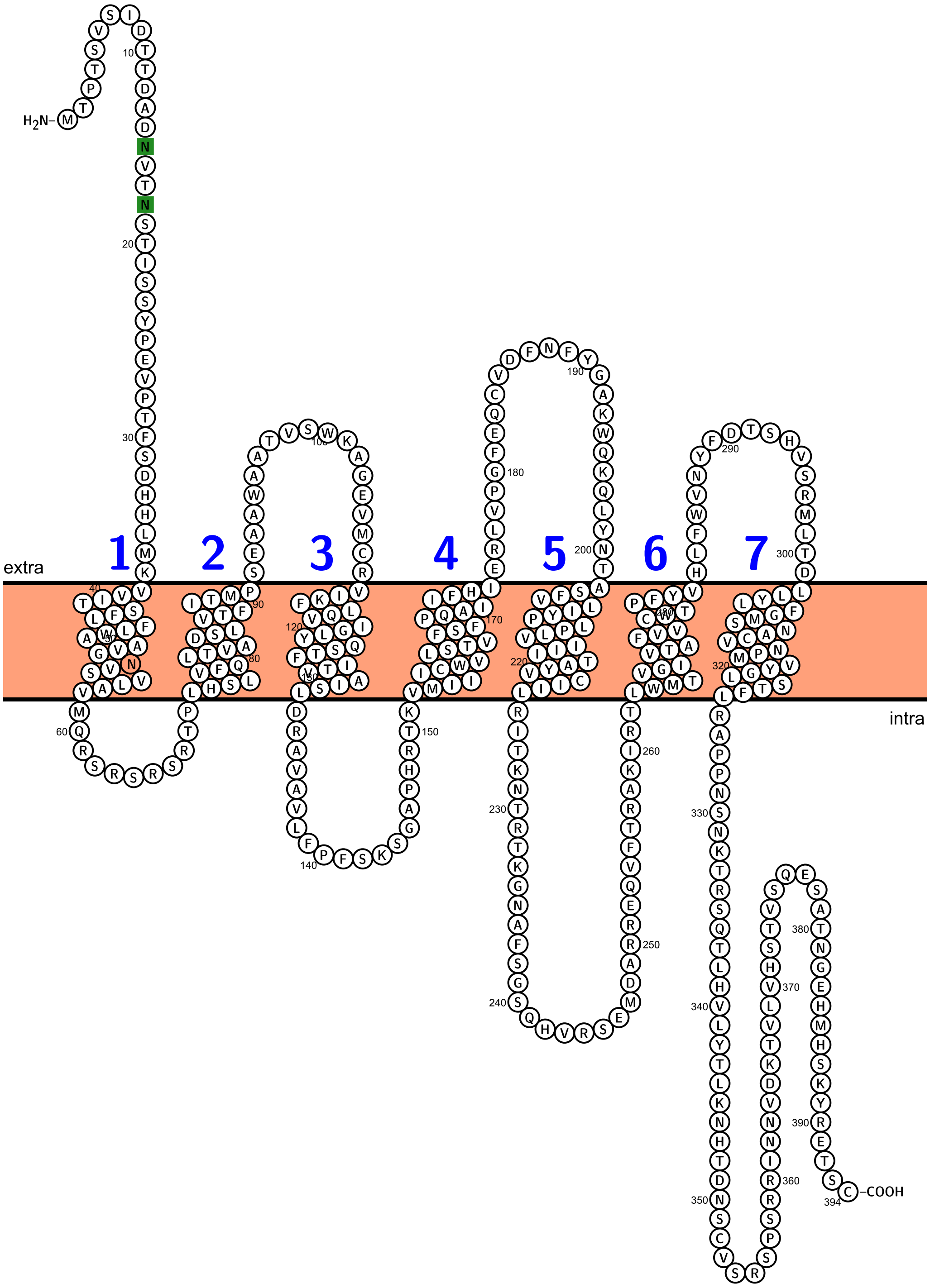

Figure S6: *In silico* prediction of the *Branchiostoma floridae* CRZR3 topology. The transmembrane domains are numbered successively in blue and the putative N-glycosylation sites are shown with green boxes.

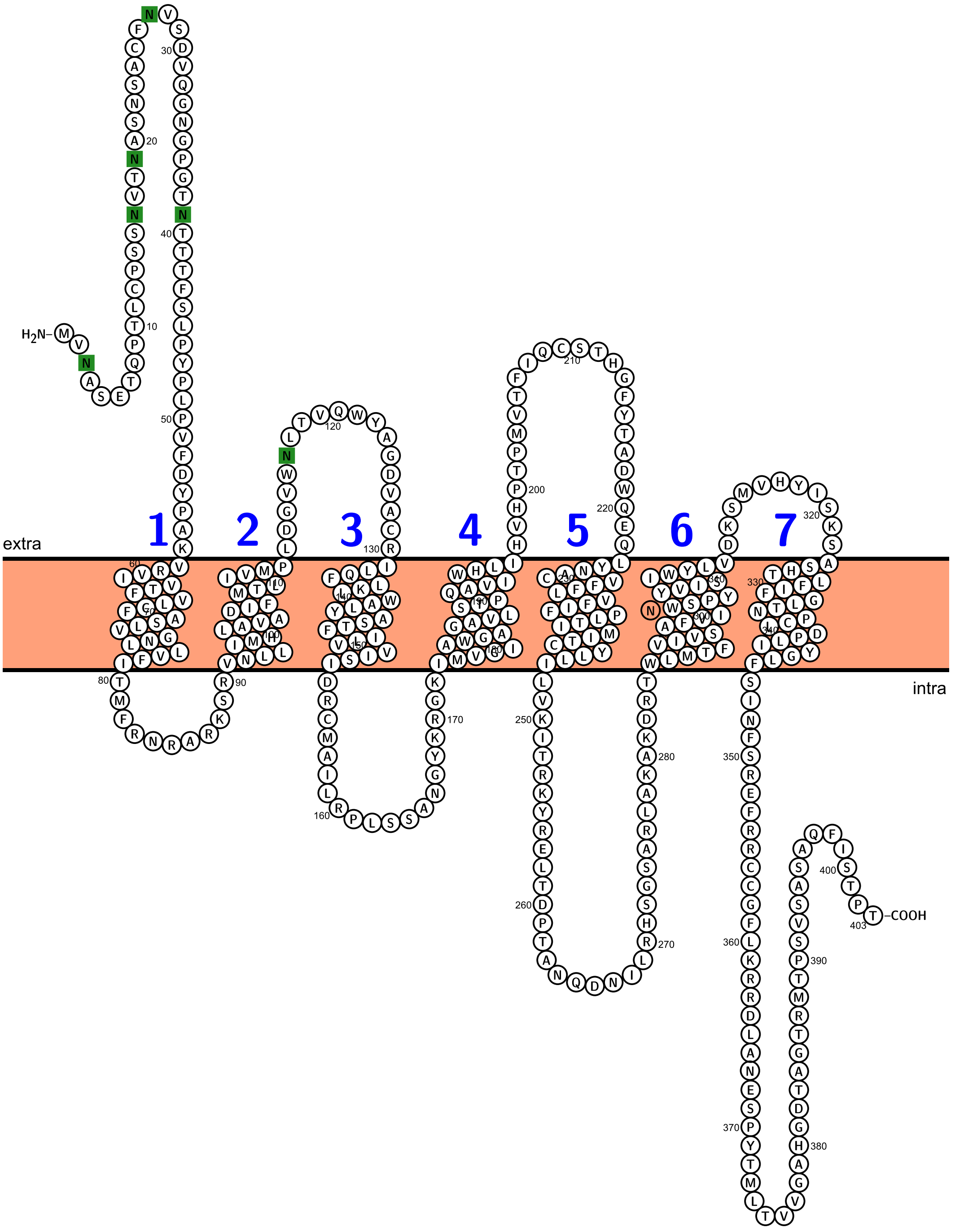

Figure S7: *In silico* prediction of the *Branchiostoma floridae* GnRHR1 topology. The transmembrane domains are numbered successively in blue and the putative N-glycosylation sites are shown with green boxes.

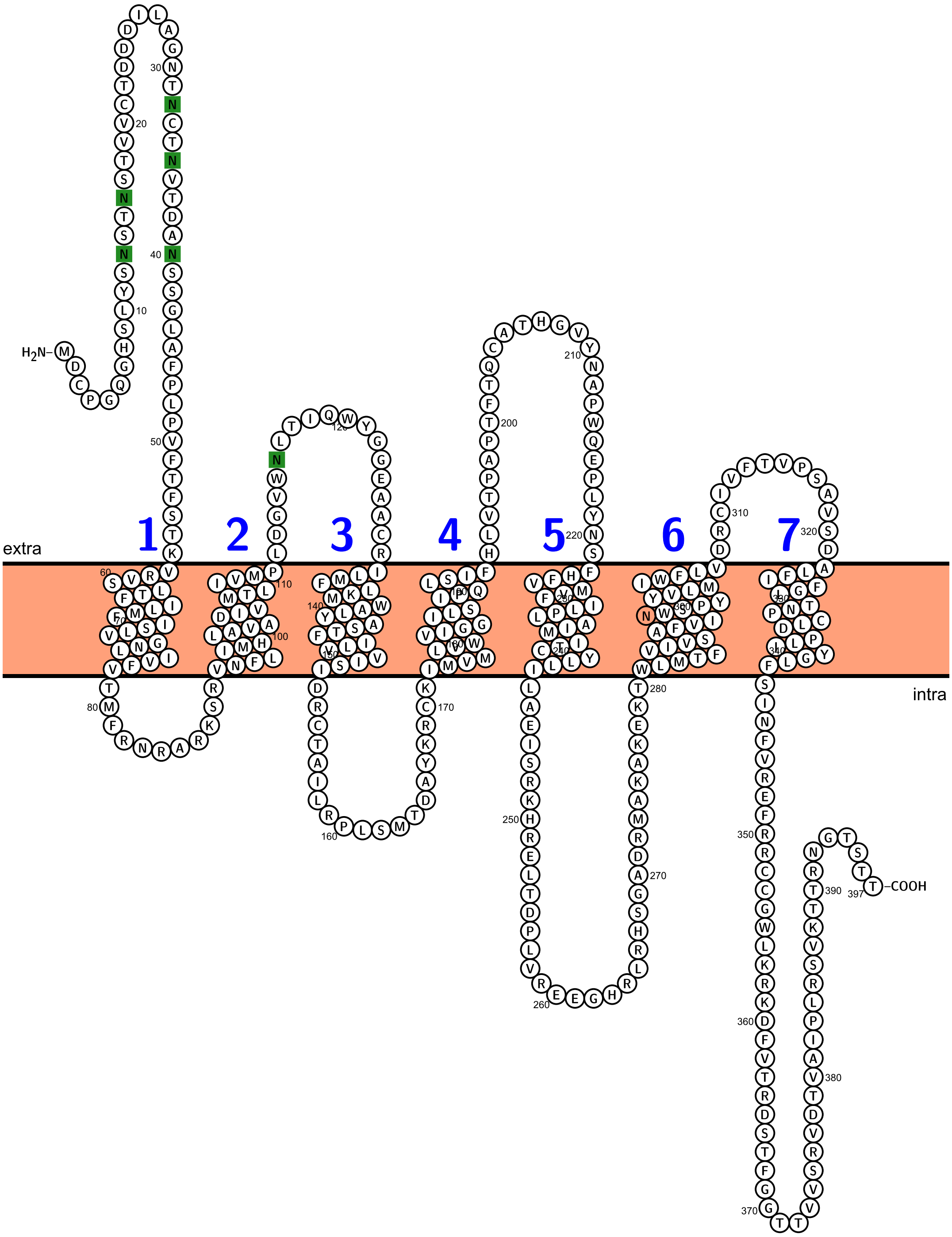

Figure S8: *In silico* prediction of the *Branchiostoma floridae* GnRHR2 topology. The transmembrane domains are numbered successively in blue and the putative N-glycosylation sites are shown with green boxes.
